# Do platelets promote cardiac recovery after myocardial infarction: roles beyond occlusive ischemic damage

**DOI:** 10.1152/ajpheart.00134.2018

**Published:** 2018-03-16

**Authors:** Tony G. Walsh, Alastair W. Poole

**Affiliations:** School of Physiology, Pharmacology and Neuroscience, University of Bristol, Bristol, United Kingdom

**Keywords:** cardiac recovery, myocardial infarction, platelets, secretion

## Abstract

Our understanding of platelet function has traditionally focused on their roles in physiological hemostasis and pathological thrombosis, with the latter being causative of vessel occlusion and subsequent ischemic damage to various tissues. In particular, numerous in vivo studies have implicated causative roles for platelets in the pathogenesis of ischemia-reperfusion (I/R) injury to the myocardium. However, platelets clearly have more complex pathophysiological roles, particularly as a result of the heterogeneous nature of biologically active cargo secreted from their granules or contained within released microparticles or exosomes. While some of these released mediators amplify platelet activation and thrombosis through autocrine or paracrine amplification pathways, they can also regulate diverse cellular functions within the localized microenvironment and recruit progenitor cells to the damage site to facilitate repair processes. Notably, there is evidence to support cardioprotective roles for platelet mediators during I/R injury. As such, it is becoming more widely appreciated that platelets fulfill a host of physiological and pathological roles beyond our basic understanding. Therefore, the purpose of this perspective is to consider whether platelets, through their released mediators, can assume a paradoxically beneficial role to promote cardiac recovery after I/R injury.

## INTRODUCTION

A classical understanding of platelet biology sees these anucleate blood cells as physiological inhibitors of bleeding from healthy blood vessels (hemostasis) but pathological instigators of occlusive events in diseased blood vessels (thrombosis) leading to ischemic damage to multiple tissues, including the heart. Antiplatelet therapies, in particular aspirin and the P2Y_12_ receptor antagonist clopidogrel, have therefore been highly successful in the primary prevention and secondary management of patients with cardiovascular disease at risk of arterial thrombosis (6, 75). However, more recently, platelets have been functionally implicated in an extensive list of nonclassical roles in the body, ranging from physiological roles in tissue regeneration, lymphangiogenesis, and vascular integrity to pathological roles in tumor angiogenesis and metastasis (22, 42, 63). Therefore, the purpose of this perspective was to explore additional roles that platelets, beyond thrombosis-mediated ischemic damage, may assume within the myocardium after myocardial infarction (MI). Our proposition is that platelets, in particular through the release of bioactive cargo, have the capacity to substantially influence phenotypic responses within infiltrating inflammatory cells and resident cardiac cells: we suggest that this allows platelets to provide paradoxically beneficial roles in cardiac recovery after MI.

## PLATELET SECRETION

Key to the functional heterogeneity of platelets is the release of a broad range of biomolecules, including over 300 proteins (comprising growth factors, chemokines, and adhesive ligands), nucleotides, and neurotransmitters. These are stored in three different classes of secretable granules [α-, dense, and lysosomal granules (7)] and are essential for classical platelet biology, acting in autocrine/paracrine manners to amplify platelet aggregation, consolidate thrombus formation, and facilitate clot remodelling. In a broader tissue context, however, these secreted ligands mediate heterocellular cross talk at the site of vascular damage and can also control the homing and differentiation of progenitor cells to facilitate tissue regeneration (56). An additional layer of complexity has been added through platelet microparticles/exosomes (PMP/Es), which represent the most abundant form of extracellular vesicles in blood (27). PMP/Es are released from activated platelets and are loaded with multiple bioactive molecules including proteins, lipids, and small noncoding mRNAs, in particular microRNAs (miRNAs): the latter have been shown to influence gene expression in a variety of different vascular cell types (32, 49).

## PLATELETS IN CARDIAC INFLAMMATION AND RESOLUTION

**Fig. 1.**
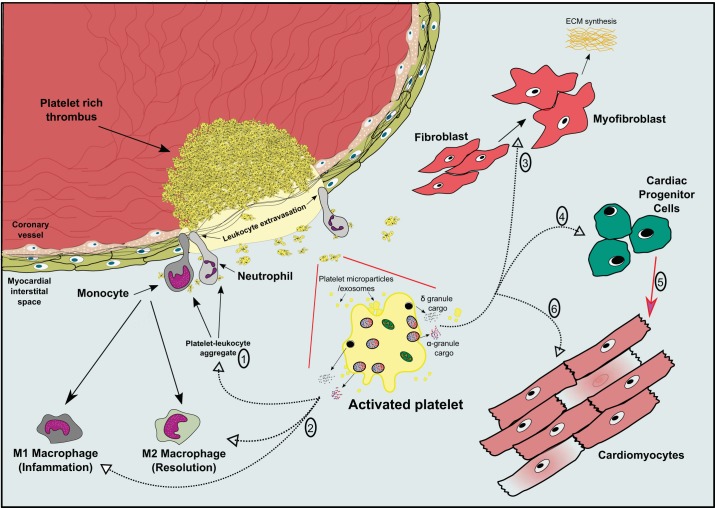
Roles for released platelet factors in inflammatory and reparative cell responses in cardiac tissue after myocardial infarction. After coronary thrombosis, activated platelets secrete granule components and release platelet microparticles/exosomes that regulate (*1*) inflammatory cell extravasation and accumulation in the myocardium and (*2*) influence the immuno-activatory responses of leukocytes, in particular neutrophils and monocytes/M1 macrophages, while also exerting immune-suppressive effects on these cells allowing M2 macrophage activity necessary for repair. Secreted platelet cargo and released platelet microparticles/exosomes can also modify resident cardiac cell responses, including (*3*) fibroblast activation and conversion to myofibroblasts to promote extracellular matrix (ECM) synthesis, while specific biomolecules known to be released from activated platelets have demonstrated roles in facilitating (*4*) cardiac progenitor cell proliferation, mobilization, and differentiation (*5*) toward cardiomyocytes to promote cardiac remodeling and repair. (*6*) Cardiomyocytes are also sensitive to released platelet molecules, which may enhance cardiomyocyte inotropic activity (adenine nucleotides and serotonin) but also provide protective/antiapoptotic signals (stromal cell-derived factor-1α/transforming growth factor-β_1_) during ischemic injury.

After MI, the heart undergoes a robust inflammatory phase lasting 3–4 days (in mice) involving upregulation of inflammatory genes and immune cell infiltration into the myocardial interstitial space to remove damaged cardiomyocytes and other cardiac cells (47). It is well known that the recruitment, transendothelial migration, and activation of immune cells such as neutrophils and monocytes into the extravascular space are facilitated by secreted platelet cargo [including chemokine (C-X-C motif) ligand 4, chemokine (C-C motif) ligand 5, and histamine] and direct interactions with proteins expressed on the platelet surface [CD62P and glycoprotein (GP)Ibα] (Fig. 1) (15, 50, 52, 62). Importantly, it has been shown that platelets, along with leukocytes, also rapidly accumulate within the infarcted myocardium, which is consistent with their tissue accumulation in other diseases, while recent studies have demonstrated a novel migratory capacity of platelets (18, 33, 37). Additionally, numerous studies demonstrating causative roles for platelets in the pathogenesis of myocardial ischemia-reperfusion (I/R) injury have shown platelet-neutrophil complexes and neutrophil accumulation in the myocardium after MI, and immune cell recruitment during this period is thought to further exacerbate the extent of myocardial damage (12, 30, 68). Indeed, platelets actively contribute to inflammatory diseases, including atherosclerosis and rheumatoid arthritis, and an excessive cardiac inflammatory response in the days after MI would cause additional cellular damage and contractile dysfunction, increasing infarct size and causing aberrant cardiac remodelling (3, 26). On the other hand, an initial, acute period of controlled inflammation is fundamental for restoring tissue homeostasis, and there are numerous studies that have reported cardioprotective and prosurvival benefits of innate immune responses after MI, including a recent study (6) demonstrating worsened cardiac function after neutrophil depletion after MI (14, 24, 65). There are therefore mixed roles for the inflammatory response in damage resolution after MI. After the acute phase, the resolution of the inflammatory cascade is crucial, allowing for an effective switch in resident- and monocyte-derived M1 macrophages toward a reparative M2 macrophage phenotype (47). In this context, it is becoming better understood that platelets actively support inflammation resolution by releasing an abundance of proresolving mediators including lipoxin A4, maresin 1, and annexin A1, which attenuate neutrophil trafficking and enhance their apoptosis (1, 2, 40, 53). The clinical potential of lipoxin A4 and annexin A1 (NH_2_-terminal derived peptide) has been recently demonstrated in vivo, by restraining inflammatory processes during cerebral I/R injury through engagement of the Fpr2/3 receptor on neutrophils (61). Interestingly, maresin 1 can induce a proresolving phenotype from platelets by suppressing their release of proinflammatory mediators (34). This proresolving capacity of platelets is further evidenced in inflammatory models demonstrating elevated levels of proinflammatory cytokines in the absence of platelets (8, 67). This raises intriguing questions regarding the pro- and anti-inflammatory properties of platelets and how they may potentially regulate inflammatory cell “switching.” For example, are there distinct stimuli that facilitate differential release/secretory patterns from platelets either to activate or suppress inflammation? Undoubtedly, it will be crucial to understand if (and how) platelets, in particular through released factors, influence innate immune responses in cardiac tissue after MI and whether they can act synergistically or independently of other resident cardiac cells to regulate immune cell activation, resolution, and the subsequent transition to a reparative response. Defining these temporal, localized signaling cues within the cardiac microenvironment after MI is undoubtedly challenging but could reveal unsuspected roles for platelets and lead to attractive novel therapeutic targets in the management of cardiac recovery after MI.

## PLATELETS AND CARDIAC FIBROSIS

In addition to infiltrating immune cells, there are other critical, resident cardiac cells that respond to the MI insult. Cardiac fibroblasts are traditionally viewed as principally responsible for the laying down of the extracellular matrix after tissue injury, a response that is essential for providing structural and functional integrity to the myocardium (59). However, this leads to the replacement of lost cardiomyocytes with collagenous scar tissue. Numerous mediators of fibroblast activation have been identified, including serotonin, transforming growth factor (TGF)-β_1_ and platelet-derived growth factor, all of which are highly enriched in platelet granules, which trigger fibroblast expansion and transdifferentiation to myofibroblasts (Fig. 1) (44, 69). Indeed, platelet-derived TGF-β_1_ has been specifically implicated in cardiac fibrosis and dysfunction after pressure overload, with a corresponding increase in plasma TGF-β_1_ derived from platelets (39). Prolonged induction of fibroblast activation leads to cardiac fibrosis and adverse remodeling, which can spread into the noninfarcted myocardium. Therefore, knowledge of endogenous inhibitory signals to control fibroblast activity would be of great therapeutic benefit as current antifibrotic strategies target activatory signals that reciprocally facilitate tissue repair (35). Thrombospondin-1 (TSP-1), a protein that is highly abundant in platelet α-granules, has been shown to negatively regulate myofibroblast density and infiltration into noninfarcted areas and also to suppress prolonged post-MI inflammatory responses (17, 66). Considering the high abundance of platelet TSP-1 relative to other tissues (29), it would be interesting to assess the direct contribution of platelet-derived TSP-1 to cardiac fibrosis with conditional knockout mice. Numerous miRNAs have been implicated in myocardial fibrosis, some of which are highly enriched in PMP/Es and can positively (miRNA-21 and miRNA-199) or negatively (miRNA-29 and miRNA-101) regulate fibrotic responses in cardiac tissue (9, 25, 45, 55). Crucially, there is experimental evidence to support the transfer of genetic material (including miRNAs) via microvesicles from other resident and nonresident cardiac cells to influence cardiac fibrosis (21, 70). Considering the abundance of PMP/Es contained by platelets, it is plausible to suggest that activated platelets could exert such heterologous cellular responses. However, given that PMP/Es (and platelet granules) possess both pro- and antifibrotic modulators, there would need to be greater complexity of control to allow fibroblasts to discriminate between opposing signals. A similar paradigm exists within the context of platelet-mediated regulation of angiogenesis and vasculogenesis, where platelets store and secrete an array of both pro- and antiangiogenic proteins from α-granules. At this time, it remains a contentious issue within the platelet field as to whether such functionally opposing proteins are differentially secreted from distinct α-granule populations or whether their release is more a stochastic process influenced by the nature of the stimulus and chemical properties of the protein (28).

## CARDIAC PROGENITOR CELL MODULATION BY PLATELETS

It has become widely appreciated that miRNAs play pivotal roles in the cardiovascular system. Endogenously, they regulate numerous vascular cells (endothelial, smooth muscle, and immune cells as well as platelets) but also resident fibroblasts, cardiomyocytes, and cardiac progenitor cells (CPCs) (55). Manipulating miRNA activity or expression is considered an attractive therapeutic target, particularly within the context of cardiac regeneration (23). Targeting CPCs to trigger their differentiation to cardiomyocytes (Fig. 1) or the “reactivation” of proliferation within cardiomyocytes to replace the cells lost during MI are some of the proposed therapeutic avenues, both of which are regulated by miRNAs but also by locally transduced mechanical and biochemical stimuli (38). Platelets also store and release a spectrum of miRNAs, including those where both positive (miRNA-199) and negative (miRNA-29) influences on cardiomyocyte cell cycle reentry have been reported (4, 16). Furthermore, miRNA-1, which is also contained within PMP/Es, has been shown to regulate CPC differentiation toward the cardiomyocyte lineage, whereas chemokine stromal cell-derived factor-1α, which is secreted from platelet α-granules, also facilitates CPC mobilization and transdifferentiation (5, 54, 58). miRNA-126, which was originally believed to be an endothelium-specific miRNA, has been confirmed by several independent groups to be one of the most abundant miRNAs in PMP/Es, and plasma levels of miRNA-126 appear to correlate with platelet activation (10, 57). Notably cardio- and atheroprotective functions have been ascribed to miRNA-126, and it has also been shown to regulate ischemia-induced angiogenesis (46, 51, 60). Currently, there is no evidence supporting PMP/E uptake by cardiac cells, but there are a number of in vitro reports demonstrating functionally relevant miRNA transfer from PMP/Es to endothelial cells and monocytes, with additional evidence supporting their release during MI (20, 32, 49). Undoubtedly, further in vitro and in vivo studies are warranted to determine if (and how) PMP/Es alter resident cardiac cell fate and whether this would confer protective or adverse consequences for cardiac recovery after MI.

## MODULATION OF CARDIOMYOCYTE FUNCTION BY PLATELETS

Outlined above are possible routes of communication between platelets and other vascular and cardiac cells. There are, however, several lines of evidence to suggest that platelets and their “secretome” can affect cardiomyocyte function independent of their role in occlusive coronary thrombosis and ischemia. From a pathophysiological context, numerous small signaling molecules that are released from platelet-dense granules (ADP/ATP, histamine, and serotonin) and thromboxane A_2_ can exert positive inotropic effects on cardiomyocytes (11). There have been a number of reports that have indirectly implicated platelets in exacerbating ischemia-induced ventricular fibrillation, while a more recent publication by Dhanjal et al. (13) provided evidence for a direct role in increasing the incidence of ventricular fibrillation (31). Interestingly, coronary artery ligation in *Unc13d^jinx^* mice, in which platelets do not secrete dense granule cargo, has no protective effect on infarct size, which argues against a significant role for dense granule secretion in the pathophysiology of myocardial injury after MI (43). On the other hand, several publications have supported a cardioprotective effect of platelet-released factors during myocardial I/R injury, including adenine nucleotides, serotonin, and thromboxane A_2,_ although these effects appear to be indirectly mediated by an intact endothelium (71–73). Similarly, recently published work from our group has further supported this cardioprotective capacity of secreted platelet factors during myocardial I/R injury (64). In this case, the protective effect on ventricular cardiomyocytes during ischemia was directly mediated by cargo actively secreted from platelet α-granules, including stromal cell-derived factor-1α and TGF-β_1_, and, importantly, this effect was substantially attenuated when platelets were pretreated with a P2Y_12_ antagonist. Given that P2Y_12_ antagonists are commonly administered to patients with MI, this observation may have implications for the clinical utility of these drugs during the early recovery phase of MI. It is also well established that platelets, through secreted molecules and PMP/Es, exert both pro- and antiapoptotic effects on different target cells (19). However, similar to the pro- and antiangiogenic capabilities of platelets, the mechanisms differentiating these opposing effects are not well understood but may reflect the relative expression levels of the respective death/survival receptors on target cells.

## CONCLUSIONS

Considering the diverse heterogeneity of the platelet secretome and PMP/Es, it is anticipated that future experimental work will uncover additional roles for released platelet factors on the various resident and nonresident cardiac cells in the acute (hours/days) and chronic (weeks) phases after MI. It is our assertion that platelets have the capacity to negate some of the deleterious consequences of coronary thrombosis by providing favorable paracrine mediators to initiate or facilitate cardiac repair processes, as directly evidenced by studies in other tissues including the liver and lungs (19, 36, 48). However, teasing out the relative contribution of platelets in an in vivo context after MI is challenging, as interfering with platelet activation and thus secretion/PMP/E release, would be likely to reduce myocardial damage after coronary thrombosis and therefore skew subsequent interpretations about roles for platelets in cardiac recovery. However, with the current, nonthrombotic model of myocardial I/R injury induced by coronary artery ligation, there have been some reports of comparable infarct rates in mice with markedly reduced platelet activation, including the study using *Unc13d^jinx^*mice mentioned above (41, 43, 74). While these responses presumably relate to the nature of the model, it would be interesting to follow up longer-term studies in conditional (PF4/GPIbα-Cre) transgenic mice with specific defects in platelet secretion or PMP/E release to monitor the post-MI responses of the various cardiac cells discussed. In conclusion, platelets are continually pushing the boundaries in terms of functional diversity, particularly through the biomolecules they release. While roles for platelets in coronary thrombosis and subsequent cardiac damage are undeniably established, there is sufficient credible evidence, as outlined in this perspective, to imply a “double-edged sword” functionality of platelets promoting cardiac recovery after MI. Furthermore, this raises intriguing questions regarding the efficacy of antiplatelet therapies, as they interfere with the release of platelet paracrine mediators and have the potential, paradoxically, to adversely impact cardiac recovery. Further work will be required to understand these complexities.

## GRANTS

This work was funded by British Heart Foundation Grant RG/15/16/31758 (to A. W. Poole).

## DISCLOSURES

No conflicts of interest, financial or otherwise, are declared by the author(s).

## AUTHOR CONTRIBUTIONS

T.G.W. and A.W.P. conceived and designed research; T.G.W. drafted manuscript; T.G.W. and A.W.P. edited and revised manuscript; A.W.P. approved final version of manuscript.
